# Prevalence of Bovine Fasciolosis, Financial Losses and Risk Factors Associated with the Disease in Lira and Gulu Districts, Northern Uganda

**DOI:** 10.1155/vmi/7757654

**Published:** 2025-03-17

**Authors:** Anthony Ogwal, Daniel Micheal Okello, Tony Aliro, David Okello Owiny, Elly Kurobuza Ndyomugyenyi

**Affiliations:** ^1^Department of Production and Marketing, Lira District Local Government, Lira, Uganda; ^2^Department of Animal Production and Range Management, Faculty of Agriculture and Environment, Gulu University, Gulu, Uganda; ^3^Department of Rural Development and Agribusiness, Faculty of Agriculture and Environment, Gulu University, Gulu, Uganda; ^4^Department of Agribusiness and Natural Resource Economics, Faculty of Agriculture and Environmental Sciences, Kabale University, Kabale, Uganda

**Keywords:** economic losses, *Fasciolosis*, liver fluke, risk, smallholder cattle farmers, Uganda

## Abstract

Bovine fasciolosis (BF) is a parasitic disease of cattle that causes significant economic impacts to cattle farmers. The physical loses include weight loss, drop in productivity, liver condemnation and mortalities. This study assessed the prevalence, financial losses and risk factors associated with the disease in Lira and Gulu Districts, northern Uganda. A cross-sectional study of 720 slaughter cattle from the abattoirs was conducted by macroscopic examination of the animals and carcasses during routine slaughter. In the farms, 120 rectal faecal samples were taken to a laboratory for *Fasciola* egg detection by simple microscopy, while risk factors were assessed by administering a questionnaire to farm owners. Prevalence of BF was highest (38%) within cattle in the age group of 1–3 years and lowest (18%) in those over 5 years. The overall BF prevalence was 48% and 26% by postmortem examination and coprology, respectively. The cattle body condition had a significant association (*p* < 0.01) with prevalence of BF, in which emaciated cattle were most affected (59%), while fat ones had the least prevalence (15%). The annual financial loss due to BF infection was USD 89,099. The major risk factors associated with the disease at the farm level were communal grazing in swampy areas (82%) and watering cattle in swamps (71%). Farms where the source of drinking water was swamp water had higher chances (*p* < 0.05) of their cattle having BF. An integrated approach using routine deworming programs, improvement of cattle management practices and control of snail intermediate hosts is recommended to effectively control the spread of BF.

## 1. Introduction

*Fasciola* are hermaphrodite parasites found in the bile ducts of most herbivorous animals, equine, pigs, rabbits and man [1]. It is common for many species of *Fasciola* to infest the same host because of livestock movement [2] as well as the distribution of its intermediate hosts, the snail [3, 4]. The prevalence of bovine fascioliasis (BF) has been reported in several African countries including Uganda [5–8]. The season of the year, type of management system and distribution of the intermediate host are among the main risk factors associated with the occurrence of BF [7, 9–11]. However, some studies reported a throughout the year occurrence of BF infection [2].

The BF is known to reduce the growth rate, fertility and productivity in terms of milk yield and draught power. Additionally, BF also leads to liver condemnation, as well as increased cost associated with administration of antihelminth. In extreme cases, the parasite causes mortality of the host livestock [12, 13]. The annual economic loss due to BF in Africa and Asia was estimated at over 200 million United States (US) dollars [14]. This is largely manifested as reduced milk yield in dairy cattle or poor feed conversion in beef cattle, leading to lighter-than-usual animals [10, 15, 16].

Studies on BF prevalence and associated losses were conducted in some abattoirs of Eastern [17], Western [5] and Central [6] regions of Uganda. However, in all these studies, risk (predisposing) factors in the farms where the animals were raised were not looked into. Similarly, the studies did not cater for the financial losses due to weight loss in the affected animals. In the main abattoirs of Lira and Gulu districts of the Northern Uganda region, there have been continued complaints by butchers incurring huge financial losses as a result of liver condemnations associated with BF (Lira and Gulu Municipal Veterinary Officers' Epidemiology Reports 2019). Despite the reports on the disease incidence in Northern Uganda, there are no dependable detailed studies conducted to establish the prevalence, actual financial loss and risk factors associated with the disease. Therefore, this study assessed the prevalence of BF, estimated the financial losses and evaluated the risk factors associated with the disease. Attention was paid to slaughter animals in the abattoirs as well as live animals in farms and the disease predisposing factors in those farms.

## 2. Materials and Methods

### 2.1. Study Area

This study was conducted in Lira (1^o^ 21′ N, 2^o^ 42″ N and 32^o^ 51″ E, 340 15″ E) and Gulu (02° 49′ 50″ N, 32° 19′ 13″ E) districts of Northern Uganda. The main abattoirs of Lira and Gulu and the subcounties of Amach, Aromo (Lira District), Paicho and Palaro (Gulu District) were used for the study. The four subcounties were selected because they supply the largest numbers of cattle to the abattoirs. Several swamps and streams cross through the two districts with low landscape of relatively uniform topography. Smallholder farmers dominate livestock production, and the major livestock kept are cattle, goats, sheep and pigs [18]. Cattle slaughtered in the two abattoirs come from within and other regions of Uganda. The abattoirs slaughter 10–20 herds of cattle per day but increases to more than double on Saturdays and Sundays, during public holidays and Christmas festive periods.

### 2.2. Study Animals

In this study, 840 heads of cattle were studied. Of these, 720 cattle were sampled from the abattoirs during routine slaughter while 120 from the farms in the four subcounties within Lira and Gulu districts. The cattle breeds used in the study were generally categorised as Ankole, Zebu and exotics (crosses). Cattle slaughtered in Gulu and Lira abattoirs are usually sourced from within the two subregions (Lango and Acholi) as well as neighbouring subregions including Teso, Karamoja, Bunyoro and Buganda.

### 2.3. Study Design and Sampling

This study followed a cross-sectional mix-method design to study the prevalence, financial losses and associated risk factors of BF in Lira and Gulu districts. It was conducted in the abattoirs and farms from February to April, 2021. This study consists of two samples. First is a sample of animals slaughtered at the abattoirs. In this study, following the literature review, 720 cattle were samples from the abattoirs of Lira and Gulu districts within a period of three months.

Each abattoir was visited 3 days a week between 5:30 AM and 7:00 AM for 3 months, making a total of 720 (120 cattle per month per abattoir) cattle for the two abattoirs. Following Howell et al. [17], the first 10 cattle to be slaughtered everyday of assessment in each abattoir were the subject of investigation. This approach to sampling that has been used in previous studies has been shown to provide accurate estimates of the prevalence of BF [19]. By selecting the first 10 cattle per day of data collection and per abattoir, the study collected equal numbers of cattle from the two abattoirs. This ensured validity and standardisation in the procedures. Prior to slaughter of the animal, critical information including age, sex, body condition score (BCS), breed as well as where the animal was brought from was recorded. Secondly, cattle farms were sampled from subcounties that supply the highest number of slaughter animals to the abattoirs for the risk analysis at the farm level. Based on preliminary analysis of the origins of the slaughtered cattle in Lira and Gulu abattoirs, the risk factors assessments were conducted in the subcounties of Aromo and Amach in Lira District and Paicho and Palaro in Gulu District. The sample size of 120 was obtained using the formula proposed by Thrusfield [20] and previously used by Mathewos et al. [21]. The sample size formula is presented in equation (1).

(1) $$\begin{array}{c} n=\frac{1.962\times P\;\exp\left(1-P\;\exp\right)}{d^{2}}, \end{array}$$

where *n* is the required sampled size, *P* exp is the expected prevalence and *d* is the absolute precision. Given that there was no recent information on the prevalence of BF in Lira and Gulu districts, the expected prevalence was assumed to be 18%, while the absolute precision was 5%. Substituting these figures in equation (1) gives a sample of 115. To account for possible nonresponse, this figure was increased to 120, which was the eventual sample size for the farm level study.

From each of the farms, the farm and farmer's characteristics were recorded, while an animal was randomly sampled from each farm for BF investigation. Multistage sampling was used to select the cattle farms in the four subcounties of Aromo and Amach in Lira District and Paicho and Palaro subcounties in Gulu District. According to District Veterinary Officers (DVOs) of the two districts, these subcounties supply the largest numbers of cattle to the abattoirs. Six parishes were randomly selected from each subcounty, making a total of 24 parishes in the four subcounties. With the assistance of the local government agricultural extension staff resident in a particular subcounty, lists of all cattle farms with at least two cattle (epidemiological units) in each of the selected parishes were used to randomly select the cattle farms. In farms where there were more than two cattle, only two were samples. This approach was adopted so as to avoid bias due to multiple animals from the same herd having similar risk factors [17]. The faecal samples from the selected cattle were taken for carpological examination. Thirty cattle were sampled from each of the four subcounties, making a total of 120 animals (60 animals per district). Cattle aged 1 year and above were selected provided their numbers were more than two in a kraal and had been in that subcounty or parish for at least 6 months.

### 2.4. Data Collection

#### 2.4.1. Assessing the Prevalence of *BF* in Cattle in the Abattoirs

Antemortem examination of the cattle was carried out shortly prior to slaughter. Cattle preslaughter inspection for any obvious signs and symptoms of BF infections was conducted when they were at rest. During this inspection, the BCS of the cattle was assessed following Nyirenda et al. [8]. Postmortem examination of the livers was performed first by visualization followed by palpation of the entire organ and finally dissection to expose the bile ducts. During the inspection, the following were recorded: date, abattoir (Lira or Gulu), origin of animal (district), BCS, age, sex, breed and the presence or absence of *Fasciola* or lesions on the liver. Following standard meat inspection guidelines and procedures [20], fluke-infested liver parts were trimmed and collected in a bucket. This included lesions, such as necrotic infarcts and tracts made by immature migrating parasites. The remaining noninfested liver parts were passed for human consumption. Where the damage was so severe, that trimming would not leave any noninfested part, and the whole live was condemned. All the condemned liver portions, together with whole livers condemned and the corresponding carcass per infected cattle, were weighed in kilograms using a weighing scale and recorded in a book.

#### 2.4.2. Assessing Prevalence of BF in Cattle in the Farms

A structured questionnaire and animal biodata form were first administered to the owner of the farm to assess the predisposing factors of BF. Fresh faecal samples were collected directly from the rectum of each animal and placed in universal bottles. Each sample was clearly labelled with animal's identification, sex, age, breed and date and place of collection. The samples were transported to Lira and Gulu districts' regional laboratory in 10% formalin in a cold box to avoid hatching and development of eggs. The eggs of *Fasciola species* were identified using concentration techniques of sedimentation [2]. The eggs of Fasciola species were identified by observing the oval shape of the eggs and/or the presence of larvae in the egg shells where the eggs have already hatched. The intensity of infection with *Fasciola* worms was determined by identification/detection and quantification of the *Fasciola* faecal egg counts into eggs per gram of faecal sample examined under a standard microscopic slide [21–23]. The total number of eggs per gram of faeces was calculated using the following equation (2):

(2) $$\begin{array}{c} \frac{\text{Eggs}}{\text{gram}}=\frac{\text{Total number of eggs counted}}{\text{Total amount }\left(\text{in grams}\right)\text{of faeces examined}}. \end{array}$$

#### 2.4.3. Assessing Financial Losses as a Result of BF Infection in Cattle

Following Arias-Pacheco et al. [24], the quantitative losses associated with liver condemnation were used to compute the direct financial loss. Indirect financial loss per infected cattle related to weight loss due to BF infection was also estimated. The financial losses due to BF were computed by calculating the value of the liver condemned and carcass weight loss due to BF by multiplying each kilogram with the corresponding market price at the time of the study for the respective study districts. The monthly average price was used for each month and for each district. To estimate the direct monetary loss associated with liver condemnation, the study adopted the formulae previously used by Nyirenda et al. [8]. The formula is as specified in equation (3):

(3) $$\begin{array}{c} LL=LC\times P_{l}, \end{array}$$

where *LL* is the total monetary loss from liver condemnation, *LC* is the quantity of the liver condemned due to BF infection per infected animal and *P*_*l*_ is the average selling price of the liver at the time of the study. In order to estimate the indirect carcass loss due to BF infection, 10% of the carcass weight was taken as an indirect loss in the carcass attributed to BF infection, following [24]. Consequently, monetary loss due to loss in the carcass was estimated by the following equation (4):

(4) $$\begin{array}{c} CL=\frac{10}{100}\times CW\times P_{C}, \end{array}$$

where *CL* is the total monetary loss per animal, *CW* is the carcass weight (in kilograms) of the slaughtered animal and *P*_*C*_ is the average selling price per kilogram of beef at the time of the study. The total loss from all infected animals would thus be obtained by summing all the individual losses attributed to each infected animal.

#### 2.4.4. Assessing the Risk Factors Associated With the Occurrence of BF in Cattle

A well-structured, close ended questionnaire was administered to 120 farmers whose cattle were chosen, and rectal faecal samples were taken. Data collected from the farms included management practices, breeds on farm, farmer's socioeconomic characteristics and information on practices such as watering, grazing system and other factors, such as the presence of snails in pastures. Additionally, data on treatments, purchases and sales of cattle were obtained. Additionally, the presence or absence of BF was assessed by sampling one or two cattle where they were more than two and evaluating its faeces for the presence of *Fasciola* eggs.

### 2.5. Data Analysis

Collected data were captured using the Microsoft Excel sheet. Data on prevalence were summarized and analysed using descriptive statistics and the chi square test. Pearson's chi-square (*χ*^2^) test was used to assess the association between prevalence of BF infection among different age groups, breeds, sex and BCS. Data obtained on financial losses were analysed using descriptive statistics. The *T*-test and analysis of variance were used to compare financial loss due to liver infection and carcass across the different categories of animal age, sex, BCS and the level of infection. The data obtained on risk factors (explanatory variables) and association between outcomes (*Fasciolosis*) were analysed using the binary logistic regression model.

## 3. Results

### 3.1. Prevalence of BF

#### 3.1.1. Prevalence of BF in Slaughtered Cattle at Gulu and Lira Abattoirs

Majority of the cattle slaughtered (83%) were of the Zebu breed. About 62% of the slaughtered cattle were bulls that were most likely between 4 and5 years of age (43%) and were of fat (44%) or lean (40%) BCS (Table 1).

Overall prevalence of BF among 720 examined cattle in both Lira and Gulu main abattoirs was 48% by postmortem liver examination (Table 2). BF prevalence was significantly higher (*p* < 0.01) in Lira abattoir (54%) than in Gulu abattoir. Similarly, BF prevalence was also significantly higher (*p* < 0.01) in cattle between 1 and3 years old. However, no significant association (*p* > 0.05) was observed between the prevalence of BF across breed, sex and body condition of the slaughtered cattle.

#### 3.1.2. Prevalence of BF in Cattle on Farms in Lira and Gulu Districts

Most dominant breed in the farms was the Zebu (80%) followed by crosses/exotic breeds (13%) (Table 3). At least 60% of the sampled cattle were cows. The dominant age group of the cattle sampled was between 4 and5 years (38%) followed by 1–3 years old (35%). Sampled cattle were classified as lean (48%), fat (33%) and emaciated (18%) according to their BCS. Further results showed that the BCS did not vary significantly (*p* ≥ 0.01) across the groups.

Overall prevalence of BF in cattle in the sampled farms was 26% being relatively higher in Lira District (Table 4). There was no significant association between districts and prevalence of BF (*p* > 0.05). However, there was significant association between the breed of cattle and prevalence with the most affected breed being the Ankole cattle, while no cross/exotic breed was found to be affected by BF at the time of the study (*p* < 0.05). Also, there was no significant association between the age (*p* > 0.05) and sex (*p* > 0.05) of cattle with prevalence of BF. The cattle body condition had a significant association with the prevalence of BF with the emaciated cattle being the most affected, while those that were considered fat had the least prevalence (*p* < 0.01)

### 3.2. Quantitative and Financial Loss Associated With BF Infection

#### 3.2.1. Quantitative Loss of the Liver and Carcass due to BF Infection

For all the 346 infected cattle from the abattoirs, 650.5 kg of liver was condemned. This implies that an average of 1.88 kg of liver was condemned for every infected animal (Table 5). Similarly, a total of 3,788.7 kg of beef was lost due to carcass weight reduction with an average of 10.95 kg per infected animal.

#### 3.2.2. Financial Losses Associated With BF in Cattle in Lira and Gulu Main Abattoirs

In computing the financial loss due to quantitative reduction in the liver and carcass, the local market prices at the time of the study were used. These were US dollars (USD) 4.4 and USD 3.9 per kilogram of the liver and beef, respectively. The average financial loss due to the liver damage per infected animal was USD 8.4, while the loss as a result of reduction in carcass weight per infected animal was USD 42.6, making a total loss of USD 51.0 (Table 6). Overall financial loss per day was USD 244.1, while the loss in 3 months of study and annually was USD 17,577.1 and USD 89,106.2, respectively.

The financial losses from condemned livers and the carcass weight reduction, as a result of BF infection in cattle, from this study were not significantly different across the abattoirs of the slaughtered infected cattle (Table 7).

#### 3.2.3. Comparing Financial Losses in Slaughtered Cattle due to BF With Breed, Sex, Age and Body Condition

The financial losses due to condemned livers and the carcass weight reduction as a result of BF infection were not significantly different across the breed, sex and age groups of the slaughtered cattle (Table 8). However, there was a significant difference (*p* < 0.01) in financial losses due to BF infection across the body condition of the slaughtered cattle. The financial loss due to condemned livers was highest in emaciated (USD 10.9) and least in fat (USD 6.5) slaughtered infected cattle. Meanwhile, the loss due to the reduction in carcass weight was highest in fat (USD 46.6) and least in emaciated (USD 39.0) infected cattle.

### 3.3. Risk Factors Associated With Occurrence of BF in Cattle in Lira and Gulu Districts

Major risk factors associated with the occurrence of BF in cattle in Gulu and Lira districts were breed of cattle, source of drinking water, farmers' awareness of existence of liver fluke and regular deworming of cattle (Table 9). Specifically, on farms where there were exotics/crosses, there were lower chances (*p* < 0.01) of cattle having BF infection as opposed to farms with Ankole or Zebu cattle breeds. Farms where the source of drinking water was swamp water had higher chances (*p* < 0.05) of cattle having BF infection, as opposed to where the water source was valley dam. There was no significant difference in risk of BF infection for all the other water sources. Farmers who were aware of the existence of the liver fluke in grazing areas or water sources were most likely (*p* < 0.01) to have cattle with BF. Consequently, as findings show, regular deworming of cattle was associated with less chances (*p* < 0.01) of BF infection in cattle. However, herd size and introduction of new animals on farm did not present any risk to BF infection.

## 4. Discussions

The categories of cattle in the study consisted of breeds such as Ankole long horn, Zebu and exotics/crosses. Both sexes were included, and all cattle were grouped into the ages of 1–3, 4–5 and those above 5 years old. They were further categorised as emaciated, lean or fat depending on the body condition. All these parameters were applied to both the slaughter cattle in the abattoirs and live cattle in the farms.

The prevalence rate of 48% obtained for the slaughter cattle in this study was much lower than the 84% reported by Joan et al. [6] in Kampala city abattoir in Central Uganda. This suggests differences in BF prevalence across time and space. For instance, the presence of liver fluke in the grazing areas is known to vary across seasons of the time [8]. Factors such as vegetation, as well as relative abundance of snails, could potentially influence variations in BF prevalence [10, 10, 17, 25, 26]. On the other hand, the prevalence rate of 48% obtained in this study was higher than the prevalence rate of 38.5% reported by Howell et al. [17] in eastern districts around the slopes of Mount Elgon and the prevalence rate of 43.7% obtained by Ssimbwa et al. [5] in Lyantonde town abattoir in south-western Uganda. This could be attributed to the variation in the climate, ecological conditions, management systems [27] and seasons (dry or wet) of the year when the studies were conducted [5, 17]. Studies have shown that the bionomic requirements for breeding *Lymnaea* snails, which are intermediate hosts required for the completion of Fasciola life cycle reach as well as development of the intramolascan stages of the flukes, often reach its peak during the wetter periods of the year [11].

In this study, male cattle formed the bulk of the sampled animals in the abattoir, while the reverse was true in the farms probably because female animals were mostly retained in the farms for reproduction and milk production. On the other hand, the male counterparts were normally sold off for slaughter immediately, and they attain slaughter weight with only a few being left for breeding purposes and ox ploughing. The female and male cattle having the same chance of being infected by the liver fluke agrees with reports from Magaji et al. [28] and Ieren et al. [29]. However, the current findings contradict previous studies [30–32] that independently reported higher incidence of liver fluke infections in females.

Local breeds had high infections than the exotic/cross breeds both in the abattoirs (48%) and on farms (26%) agreeing with the findings of Aliyu et al. [19] who reported a 29% prevalence in the local breeds and no infection in the exotic/crosses. However, Ssimbwa et al. [5] reported a lower prevalence of the liver fluke infection in the local breed (25%) as opposed to the exotics/crosses (54.8%). In the current study, the higher prevalence obtained from the local breed could be due to the predominance of this breed in the study area. Due to the decreasing grazing lands, farmers are compelled to graze local breeds in swampy areas and wetlands that could be heavily infested with snail, the intermediate hosts of the liver fluke. This is especially in the dry season, where the upland pasture becomes so scarce. On the other hand, most exotics/crosses are given routine healthcare, for instance, regular deworming, and are kept under zero grazing (intensive) or semi-intensive management, where planted grasses and legumes are cut and fed to them. Hence, they are less prone to getting liver fluke infections.

The body condition of cattle is strongly linked to the development of Fasciola infection. Emaciated cattle are for instance more at risk of development of BF infection. Similar observations were made by Asrese and Ali [33]. Emaciation resulting from malnutrition or health challenges is associated with reduced resistance to parasitic infections. This is because infected cattle give priority to the reversal of the pathophysiological consequences of parasitism over the body functions and growth [19]. Age had significant effect on the prevalence of BF, being higher in young animals (38%) than the adults. This suggests that older cattle could have developed better mechanisms of fighting parasitic infections. Similar findings were reported by previous studies that argued that the decrease in prevalence as age increases is the result of acquired immunity in older animals, which is manifested by the humeral immune response and tissue reaction in the bovine liver due to previous challenges [22, 34]. The increased resistance against BF (low prevalence) with age is also related to the high level of tissue reaction where liver fibrosis impedes the passage of immature flukes, acquired thickening, stenosis and calcification of bile ducts, rendering the liver an unfavourable site for adult parasites [35]. Moreover, inverse correlation of prevalence and age of cattle have also been previously reported [5, 26, 36].

This study revealed that the financial loss from the carcass weight reduction was of considerable amount as compared to liver condemnation and should not be neglected. The total annual financial loss of USD 89,106.2 due to *Fasciolosis* in this study was lower than the USD 64,289,919.4 loss reported by Joan et al. [6] in Kampala city abattoir, Uganda. This difference is largely due to the number of animals involved in the studies. Similarly, the financial loss of USD 8.4 per liver condemned was higher than the USD 4.6 reported by Ssimbwa et al. [5] in Lyantonde town abattoir, Uganda and the USD 2.1 reported by Arias-Pacheco et al. [24] in the Peruvian Andes. The financial loss due to liver condemnation was only 16.5% of the total losses for BF infection. This suggests that, the indirect loss from BF infection due to reduction in the carcass weight was more than five times the loss from liver condemnation per infected cattle. A study by Arias-Pacheco et al. [24] reported indirect losses of up to 10 times the loss from liver condemnation. This difference is attributed to intensity of the liver damage due to BF infection.

The total direct and indirect losses reported in this study provide further evidence that BF is an important parasitic disease, causing great loss of revenue due to condemnation of affected livers and weight loss of the affected cattle. Additionally, condemnation of large quantities of livers as well as reduction in carcass quantity leads to their scarcity with associated price increases [37, 38], hence making it more expensive to consumers.

In this study, the high risk of acquiring *Fasciolosis* when animals were grazed communally in wet swampy areas agrees with the findings of earlier studies [10, 26]. This is not surprising considering that snails prefer swampy areas for survival. Routine deworming of cattle reduced the risk of acquiring BF by about five times, implying that improving management practices and strategic deworming programs helps in the control of the disease in northern Uganda. Freely and communally grazed pastures are usually contaminated by cattle dung, thus increasing the risk of infection in extensively managed herds. This risk is especially higher in grazing pastures with abundance of snails [7, 39]. This may be a possible reason for the higher prevalence of the disease observed among extensively managed cattle.

The low awareness of BF and its occurrence among farmers in the current study has a bearing on the adoption of better practices that limit the spread of the disease. This is because such farmers could have encountered liver fluke (the intermediate host of BF) in the grazing but did nothing to avoid their animals being affected. The majority of farmers were unable to link the development stages of the BF with the intermediate hosts, hence making it difficult to control the transmission cycle. Adoption of better practices that limit risks of BF is highly influenced by awareness of the transmission cycle [36]. Farmers' knowledge or awareness of existence of BF, mode of transmission and cure are important for cattle-keeping communities to get rid of the disease [36]. Despite the low awareness of BF among farmers, the routine practice of deworming cattle has been useful in limiting the transmission of the disease. Male farmers were more knowledgeable about the disease condition compared to females, which could be attributed to the fact that in the study area, cattle rearing and healthcare are more of the responsibility for men. In agreement with earlier studies [11, 15, 25, 26], most farmers in this study who routinely dewormed their cattle using drugs demonstrated the success achieved by chemotherapy. However, some farmers dewormed their animals not necessarily because of their awareness about the specific dangers of Fascioliasis but as a way of controlling worm infestation generally. This study revealed that there was no significant variation in prevalence of *Fasciolosis* between herd sizes probably because of the similar communal grazing system that gave equal exposures to contaminated grass by cattle during grazing.

## 5. Conclusions

BF prevalence in cattle was high (48%) in abattoirs and (26%) in farms in Lira and Gulu districts, northern Uganda, and the awareness of the disease among farmers was low. The disease is associated with significant financial losses as a result of condemnation of edible liver tissues and weight loss. The risk of cattle acquiring BF was mainly influenced by communal grazing and watering cattle in swamps, the two being highly risky compared to other factors. Effective control of the disease requires an integrated control approach using routine deworming programs, improvement of cattle management practices and control of snail intermediate hosts.

## Figures and Tables

**Table 1:** Characteristics of slaughtered cattle in Lira and Gulu main abattoirs.

Variable	Category	Number of cattle (*n* = 720)	Percentage
Breed	Ankole	95	13.2
	Exotic/crosses	29	4.00
	Zebu	596	82.8

Sex	Male	445	61.8
	Female	275	38.2

Age (years)	1–3	176	24.4
	4-5	311	43.2
	> 5 years	233	32.4

Body condition	Emaciated	118	16.4
	Fat	314	43.6
	Lean	288	40.0

**Table 2:** Prevalence of BF in Lira and Gulu main abattoirs in relation to breed, sex, age and body condition.

Variable	Category	Number of infected cattle	% prevalence	Chi-square	*p* value
Abattoir (*n* = 360)	Gulu	151	41.9	10.8	0.001
	Lira	195	54.2		

Breed	Ankole	45	47.4	1.30	0.523
	Exotic/crosses	11	37.9		
	Zebu	290	48.7		

Sex	Male	221	49.7	1.21	0.272
	Female	125	45.5		

Age (years)	1–3	96	54.6	16.1	< 0.001
	4–5	163	52.4		
	> 5 years	87	37.3		

Body condition	Emaciated	66	55.9	3.62	0.164
	Fat	144	45.9		
	Lean	136	47.2		

Overall prevalence	—	346	48.1	—	—

**Table 3:** Characteristics of cattle in the farms in Lira and Gulu districts.

Variable	Category	Frequency	Percentage
Breed	Ankole	9	7.5
	Exotic/crosses	15	12.5
	Zebu	96	80.0

Sex	Male	48	40.0
	Female	72	60.0

Age group	1–3 years	42	35.0
	4–5 years	45	37.5
	Over 5 years	33	27.5

Body condition score	Emaciated	22	18.3
	Fat	40	33.3
	Lean	58	48.3

**Table 4:** Prevalence of BF in cattle in the farms of Gulu and Lira districts in relation to breed, sex, age and body condition.

Variable	Category	Number of infected cattle	% prevalence	Chi-square	*p* value
District	Lira	19	32.0	2.13	0.144
	Gulu	12	20.0		

Breed	Ankole	4	44.4	7.12	0.029
	Exotic/crosses	0	0.00		
	Zebu	27	28.1		

Sex	Male	13	27.1	0.065	0.798
	Female	18	25.0		

Age (years)	1–3	16	38.0	5.19	0.075
	4-5	8	18.0		
	> 5	7	21.0		

Body condition	Emaciated	13	59.0	16.0	< 0.001
	Fat	6	15.0		
	Lean	12	21.0		

Overall prevalence	—	31	25.8	—	—

**Table 5:** Quantity of the liver and carcass from the infected cattle.

Tissue affected	Mean ± SD	Minimum	Maximum
Quantity of the liver condemned (kg)	1.88 ± 1.29	0.100	7.00
Reduction in carcass weight (kg)	10.95 ± 1.91	7.10	22.0

**Table 6:** Financial loss from liver condemnation and reduction in carcass weight as a result of BF in cattle.

Financial loss per animal (UGX)	Mean ± SD	Minimum	Maximum
Financial loss from the condemned liver	30,083 ± 20,693	1600	112,000
Financial loss from carcass weight reduction	153,258 ± 26,735	99,556	311,111
Total financial loss	183,341 ± 24,154	116,889	312,711

*Note:* The exchange rate at the time of the study was 1 USD = 3596 UGX.

**Table 7:** Comparing financial loss from the BF infection by abattoir.

Lost portion	Lira	Gulu	*t*-stat	*p* value
	Mean ± SD	Mean ± SD		
Liver condemnation	30,954 ± 21,127	28,959 ± 20,131	0.889	0.375
Reduction in carcass weight	153,880 ± 28,698	152,455 ± 24,035	0.491	0.623

*Note:* The exchange rate at the time of the study was 1 USD = 3596 UGX.

**Table 8:** Financial losses due to BF in relation to breed, age and body condition.

Variable	Financial loss from condemned livers (UGX)	Financial loss from carcass weight reduction (UGX)
Breed		
Ankole	23,236 ± 18,193	154,138 ± 32,474
Exotic/crosses	32,436 ± 23,352	170,970 ± 53,807
Zebu	31,057 ± 20,819	152,450 ± 24,080
*F*-value	2.89	2.60
*p* value	0.057	0.076
Sex		
Female	28,525 ± 20,144	151,710 ± 23,658
Male	30,965 ± 20,990	154,134 ± 28,343
*t*-stat	1.054	0.810
*p* value	0.293	0.419
Age (years)		
1–3	28,233 ± 17,918	151,764 ± 23,602
4-5	30,577 ± 20,895	153,933 ± 28,170
> 5	31,200 ± 23,133	153,642 ± 27,454
*F*-value	0.555	0.210
*p* value	0.574	0.811
Body condition		
Emaciated	39,345 ± 21,674^a^	140,377 ± 22,582^a^
Fat	23,450 ± 19,389^b^	167,438 ± 26,370^b^
Lean	32,612 ± 19,352^c^	144,495 ± 21,716^c^
*F*-value	16.4	44.0
*p* value	< 0.001	< 0.001
Intensity of infection		
Mild	13,811 ± 10,924^a^	164,691 ± 25,920^a^
Moderate	26,549 ± 12,385^b^	158,926 ± 22,162^b^
Severe	57,060 ± 14,661^c^	128,749 ± 18,574^c^
F-value	296	69.1
*p* value	< 0.001	< 0.001

*Note:* For each comparison category, means with different superscripts are significantly different at *p* = 0.05; the exchange rate at the time of the study was 1 USD = 3596 UGX.

**Table 9:** Binary logistic regression model analysis of risk factors associated with BF infection in cattle in Lira and Gulu districts.

Explanatory variable		Estimate (SE)	OR	95% CI	*p* > *z*
Sex of farmer (1 = female)		1.361 (0.775)	3.901	0.854–17.821	0.079

Age (above 40 = base)	Less than 30 years	−1.199 (0.784)	0.302	0.065–1.403	0.126
	31–40 years	−0.958 (0.601)	0.384	0.118–1.245	0.111

Breed on farm (ankole = base)	Crosses	−6.441 (2.469)	0.002	0.001–0.201	0.009
	Zebu	−2.226 (1.450)	0.108	0.006–1.851	0.125

Herd size (number)		0.182 (0.101)	1.199	0.984–1.462	0.072

New animal introduced (1 = yes)		−1.515 (1.099)	0.220	0.026–1.894	0.168

Source of drinking water for animals (valley dam = base)	Swamp	2.414 (1.077)	11.179	1.355–92.230	0.025
	Well	0.897 (0.993)	2.452	0.350–17.186	0.367
	Stream	1.624 (0.970)	5.075	0.758–33.970	0.094
	Borehole	1.299 (1.056)	3.666	0.463–29.023	0.218

Dry season grazing area (communal pasture base)	Swamp	−0.684 (0.806)	0.504	0.104–2.448	0.396
	Garden	−0.829 (1.047)	0.436	0.056–3.399	0.428

Wet season grazing area (communal pasture base)	Swamp	1.323 (1.194)	3.753	0.362–38.944	0.268
	Garden	1.898 (1.113)	6.674	0.754–59.083	0.088

Aware of liver fluke (1 = yes)		2.081 (0.766)	8.013	1.786–35.949	0.007

Deworms animals (1 = yes)		−3.701 (0.736)	0.025	0.006–0.105	< 0.001

Constant		−1.941 (2.589)	0.144	0.001–22.945	0.453
Number of observations		120			

Wald chi^2^ (17)		38.20			

Prob. > chi^2^		0.0023			

Log likelihood		−46.098			

Pseudo *R*^2^		0.4386			

## Data Availability

The datasets used in this study are available from the corresponding author upon reasonable request.
